# Association between micro particle-tissue factor activity, factor VIII activity and recurrent VTE in patients with acute pulmonary embolism

**DOI:** 10.1007/s11239-015-1180-z

**Published:** 2015-02-11

**Authors:** Judith Kooiman, Paul L. den Exter, Inci Kilicsoy, Suzanne C. Cannegieter, Jeroen Eikenboom, Menno V. Huisman, Frederikus A. Klok, Henri H. Versteeg

**Affiliations:** 1 Department of Thrombosis and Haemostasis, Leiden University Medical Center, C7-Q, P.O. Box 9600, 2300 RC Leiden, The Netherlands; 2 Department of Clinical Epidemiology, Leiden University Medical Center, Leiden, The Netherlands

**Keywords:** Pulmonary embolism, Recurrent venous thromboembolism, Micro particle-tissue factor, Factor VIII activity, Epidemiology

## Abstract

Studies on the association between microparticle expressing tissue factor (MP-TF) activity, FVIII activity (FVIII:C) and recurrent VTE yielded inconclusive results. We studied these associations in patients diagnosed with acute pulmonary embolism. Plasma levels of MP-TF and FVIII activity were measured in 277 patients with a first and 72 patients with a recurrent VTE. All patients were categorized based on the quintiles of MP-TF and FVIII activity in those with a single VTE. For both markers, odds ratios (ORs) for recurrent VTE were computed using patients in the lowest quintile as a reference group. No association was observed between MP-TF activity and recurrent VTE, with an OR of 1.4 (95 % CI 0.7–2.9) in the highest quintile of MP-TF activity. Compared with the reference group, patients in the highest quintile of FVIII:C were at increased risk of recurrent VTE, OR 4.2 (95 % CI 1.4–12.2). MP-TF activity was not associated with recurrent VTE whereas high FVIII:C levels were associated with a 4-fold increased risk of VTE recurrence. Future prospective studies are necessary to explore the potential of FVIII:C as a tool for risk stratification, either by itself or in combination with other pro-thrombotic markers.

## Introduction

Venous thromboembolism (VTE) is the third most common cardiovascular disease, with an incidence rate of 1–2 per 1000 patient years [1, 2]. After a first event, the 5-years cumulative risk of recurrent VTE is estimated to be 25 %, with a case-fatality rate of 11 % [3, 4]. Treatment with anticoagulants is highly effective in reducing the risk of recurrent VTE [5, 6], but this comes at the cost of an increased risk of major bleeding events [7]. Understanding of the mechanism of recurrent VTE and identification of patients at high risk of recurrences are therefore the essential step in determining the optimal duration of treatment. They requires understanding of factors contributing to the recurrence of VTE, such as clinical variables and laboratory markers.

Microparticle expressing tissue factor (MP-TF) has recently been proposed as a potential key player in the development of VTE [8]. Tissue factor is the main initiator of the coagulation cascade and MP-TFs are shed into the circulation by cells (mainly platelets and to a lower extent monocytes) during activation or apoptosis. The influence of MP-TF and MP-TF activity on the occurrence of VTE has mainly been studied in animal models [9–12]. So far, only two small clinical studies analyzed the association between MP-TF or MP-TF activity and the risk of a first or recurrent VTE [13, 14]. Therefore, the primary aim of our study was to assess the association between MP-TF activity and the risk of recurrent VTE.

Additionally, several studies have been performed on the association between FVIII activity (FVIII:C) (another potential key player in the development of VTE) and recurrent VTE, but they yielded contradictory results [15–20]. As FVIII:C was measured in our study population for other purposes, we were also interested in studying the association between FVIII:C and recurrent VTE.

## Methods

We performed a post hoc analysis using data of patients included in a cohort study that aimed to evaluate the incidence of chronic thromboembolic pulmonary hypertension (CTEPH) after a diagnosis of acute pulmonary embolism (PE). Details of this cohort study have been described in detail previously [21, 22]. In brief, patients diagnosed with acute PE between 2001 and 2007 in an academic and its affiliated teaching hospital (i.e. Leiden University Medical Center, Medical Center Haaglanden, both the Netherlands) were asked to participate in a CTEPH screening program. The CTEPH screening program was executed between July 2007 and January 2009. The diagnosis of acute PE was confirmed by detection of an intraluminal filling defect on pulmonary angiography or computed tomography pulmonary angiography, a high probability ventilation perfusion scintigraphy (VQ-scan), or an intermediate probability VQ-scan in combination with deep vein thrombosis diagnosed by ultrasonography. Patients were excluded if they had known CTEPH or pulmonary hypertension of other etiology at time of screening, if they were geographic inaccessible (living outside the Netherlands), if they were unavailable for objective testing, or recently had undergone echocardiography ruling out pulmonary hypertension. For this post hoc analysis, we included all study patients from the original study cohort except for those without available lab samples.

All patients were treated for acute PE according to local clinical practice, with unfractionated heparin or low-molecular-weight-heparin for at least 5 days after PE diagnosis followed by a period of at least 6 months of vitamin K-antagonist treatment.

The protocol of the study was approved by the Institutional review board of the two hospitals and all patients provided written informed consent.

### Screening program

**Fig. 1:**
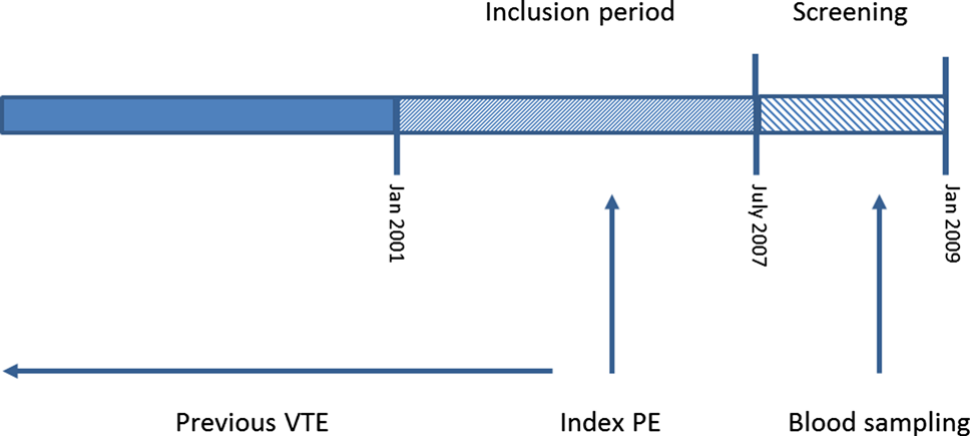
Study design

Patients were invited to the vascular medicine outpatient clinic for a single study visit. This visit was planned at least 1 year after index PE, to rule out the initial effect of acute PE on the test results (Fig. 1). During the study visit, a physical examination, echocardiography, pulmonary function and exercise tests were performed. In addition, blood samples were drawn. Information on comorbidity, diagnostic management, previous episodes of VTE, and the clinical course after index PE was obtained from anamnesis and medical records.

### Outcome

The primary outcome for this post hoc analysis was recurrent VTE. Follow-up for the endpoint of recurrent VTE ended at time of index PE (Fig. 1). VTE recurrences that occurred after the index PE were not taken into account to limit the effect of potential survival bias on the association between MP-TF activity or FVIII:C and recurrent VTE. Recurrent VTE was therefore defined as a history of VTE at time of index PE. Previous episodes of superficial DVT or VTE at unusual sites (i.e. retinal vein occlusion or portal vein thrombosis) were not taken into account for the diagnosis of recurrent VTE. Medical records of study patients at both hospitals were searched for information regarding the diagnosis and treatment of previous VTE episodes.

### Blood sampling

Citrate samples (5 ml) were taken by a single vena puncture. A tourniquet was used prior to sampling, but removed at time of blood drawing. Citrate samples were centrifuged for 10 min at 2700 g at 18 °C. Samples were stored at −80 °C within 1 h after sampling.

### MP-TF activity assay

MP-TF activity was measured in citrate plasma using a TF-activity assay as described before, with a detection limit of 0.5 fM Xa/min [23]. For the measurement of MP-associated TF activity (MP-TF activity), MPs were washed extensively to reduce contamination with plasma proteins (0.5 % in the final MP preparation); 500 μl of plasma was centrifuged at 18,890×*g* for 30 min and subsequently, 450 μl was removed. 900 μl PBS/0.32 % citrate was mixed with the pellet and the centrifuge step was repeated. Finally, the pellet was washed with 450 μl of PBS/0.32 % citrate and resuspended in 70 μl PBS/citrate. MP-TF activity measurements were then performed in triplicates; one sample of the triplicates functioned as the experimental sample, one sample as a control by leaving out FVII, and one sample as another control by adding 125 μg/ml TF blocking antibody. Twenty-five μM dioleoylphosphatidylserine (DOPS):dioleoylphosphatidylcholine (DOPC) (10:90) vesicles were incubated in a buffer consisting of 10 mm Hepes, pH 7.45, 137 mm NaCl, 4 mm KCl, 5 mg mL^−1^ ovalbumin, 50 nm hirudin and 6 mm CaCl_2_ for 15 min. To 100 μL of this solution 20 μL MP-suspension was added. 40 μL 5 nm FVII (or buffer) was added. After 10 min, 25 μL 2.5 mm S2765 was added and the reaction started by adding 40 μL 250 nm FX. The absorbance at 405 nm (expressed in mAbs) was recorded for 90 min using a Spectra Thermo Tecan ELISA reader, and plotted as a function of time (t) and, after correction for the absorbance in the absence of FVII or presence of the TF blocking antibody, as a function of t2. The slope of the latter curve is a measure for the rate of FXa generation and expressed as mAbs min^−2^ or as fM FXa min^−1^.

### Factor VIII measurements

Plasma factor VIII activity (FVIII:C) was measured by a one-stage APTT-based clotting assay using immunodepleted FVIII-deficient plasma and automated APTT (BioMerieux, Boxtel, the Netherlands).

### Statistical analysis

All patients included in this post hoc analysis were categorized as r cases (i.e. patients diagnosed with recurrent VTE at time of index PE) or controls (i.e. those with a first VTE at time of index PE). Mean MP-TF and FVIII activity values were compared between cases and controls using Mann–Whitney *U*-test. Logistic regression analyses were used to study whether MP-TF en FVIII activity were associated with recurrent VTE. Patients were categorized based on the quintiles of MP-TF and FVIII activity levels in controls. Using patients in the lowest quintile of MP-TF and FVIII activity as a reference group, odds ratios (ORs) for recurrent VTE were reported as crude values and as values adjusted for age, gender, and active malignancy at time of sampling. OR were not adjusted for VKA use at time of blood sampling as VKA therapy was not regarded as a confounder in the analysis on the association between MP-TF or FVIII activity and recurrent VTE. Sensitivity analyses were performed stratified for time between index PE and lab sampling, to study its effect on the association between MP-TF activity, or FVIII:C and the risk of VTE recurrences. In addition, we analyzed whether patients with FVIII:C > 1.66 IU/ml (i.e. the cut-off previously used by Christiansen, et al.) were at increased risk of recurrent VTE [15].

All analyses were performed using SPSS, version 20 (IBM Corp, Armonk, New York).

## Results

### Population

**Fig. 2:**
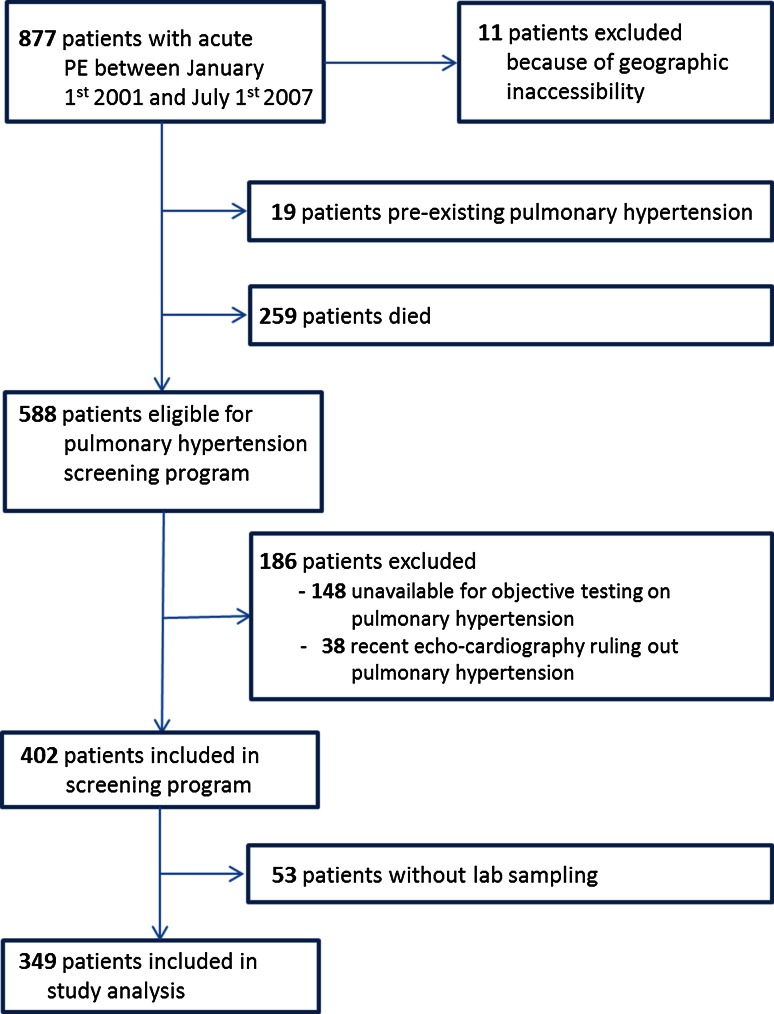
Flow chart

We identified 877 patients diagnosed with acute PE between 2001 and 2007. A total of 11 patients emigrated and were therefore unavailable for enrollment. Additionally, 259 patients died before 2007 and 19 patients were already diagnosed with pulmonary hypertension, leaving 588 patients eligible for inclusion (Fig. 1). Of those, 186 were excluded because they were either unavailable for screening or had recently undergone echocardiography ruling out CTEPH. As citrate samples were lacking for 53 patients due to logistical reasons or patient refusal, our final study population comprised 349 of the 402 (87 %) patients that entered the CTEPH screening program (Fig. 2).

The study population consisted of 72 cases (20.6 %, i.e. those with recurrent VTE) and 277 controls (79.4 %, i.e. those with a first event at time of index PE). Median time between index PE and the screening visit was 44 months (2.5–97.5 percentiles 14–83 months). Patient characteristics of cases and controls at time of screening are reported in Table 1.

**Table 1:** Characteristics of included patients at time of registration

	Cases	Controls
*N*	72	277
Age (mean, SD)	58.8 (15.2)	53.5 (15.4)
Male gender	48 (66.7)	128 (46.2)
Provoked PE^a^	32 (44.4)	192 (69.3)
Body mass index (kg/m^2^, mean, SD)	29.0 (5.1)	28.1 (5.5)
Active malignancy	7 (9.7)	31 (11.2)
COPD	9 (12.5)	22 (7.9)
VKA use	51 (70.8)	47 (17.0)
History of stroke	8 (11.1)	37 (14.1)
Peripheral artery disease	0 (0.0)	5 (1.8)
Family history of VTE	28 (38.9)	91 (32.9)

Data are presented as *n*,  % unless stated otherwise

*SD* standard deviation, *PE* pulmonary embolism, *COPD* chronic obstructive pulmonary disease, *VTE* venous thromboembolism, *IQR* interquartile range, *VKA* vitamin K-antagonist

^a^Index episode of PE

### MP-TF activity

Median MP-TF activity was 32.2 fM Xa/min (2.5–97.5 percentile 0.0–181.7 fM Xa/min) for cases and 19.3 fM Xa/min (2.5–97.5 percentile 0.00–192.5 fM Xa/min) for controls (*p* = 0.12). Controls in whom the index event was unprovoked had a median MP-TF activity of 13.6 fM Xa/min (2.5–97.5 percentile 0.0–230.2 fM Xa/min) compared with 21.8 fM Xa/min (2.5–97.5 percentile 0.0–163.2 fM Xa/min) in controls in whom the index event was provoked by a thromboembolic risk factor (*p* = 0.47). No evidence of an association was observed between MP-TF activity and the risk of recurrent VTE, comparing the different quintiles with the reference group (i.e. those in the lowest quintile of MP-TF activity), with a non-significant crude OR of 1.3 (95 % CI 0.6–2.7) in the highest quintile of MP-TF activity (Table 2). A regression analysis stratified for time (i.e. 0–2, 2–4, and >4 years) between index PE and study visit showed no effect of the duration of this time period on the association between MP-TF activity and recurrent VTE (Table 2).

**Table 2:** Microparticle-tissue factor activity in cases and controls

Time to index PE	Percentile	MP-TF activity (fM Xa/min)	Cases (*N* = 72)	Controls (*N* = 277)	OR^a^ (95 % CI)	OR^b^ (95 % CI)
All patients	0–20	<0.9	15	55	Ref	Ref
	20–40	0.9–11.9	7	56	0.5 (0.2–1.2)	0.4 (0.2–1.2)
	40–60	11.9–29.4	9	55	0.6 (0.2–1.5)	0.6 (0.2–1.5)
	60–80	29.4–65.1	21	57	1.4 (0.6–2.9)	1.3 (0.6–2.9)
	80–100	>65.1	20	54	1.3 (0.6–2.7)	1.3 (0.6–2.8)
0–2 years	0–20	<0.9	2	15	Ref	Ref
	20–40	0.9–11.9	2	14	0.9 (0.1–7.6)	1.1 (0.1–9.7)
	40–60	11.9–29.4	2	14	0.9 (0.1–7.6)	1.1 (0.1–9.4)
	60–80	29.4–65.1	4	15	1.7 (0.3–11.1)	1.5 (0.2–10.3)
	80–100	>65.1	10	2	1.3 (0.2–10.9)	1.0 (0.1–9.1)
2–4 years	0–20	<0.9	8	24	Ref	Ref
	20–40	0.9–11.9	2	17	0.4 (0.1–1.9)	0.4 (0.1–2.1)
	40–60	11.9–29.4	4	20	0.6 (0.2–2.3)	0.5 (0.1–2.0)
	60–80	29.4–65.1	7	18	1.2 (0.4–3.8)	1.0 (0.3–3.9)
	80–100	>65.1	9	21	1.1 (0.4–3.4)	0.9 (0.3–3.0)
>4 years	0–20	<0.9	5	18	Ref	Ref
	20–40	0.9–11.9	3	25	0.4 (0.1–2.0)	0.5 (0.1–2.3)
	40–60	11.9–29.4	3	21	0.5 (0.1–2.5)	0.5 (0.1–2.7)
	60–80	29.4–65.1	10	24	1.5 (0.4–5.2)	1.8 (0.5–6.6)
	80–100	>65.1	9	23	1.4 (0.4–4.9)	1.8 (0.5–6.9)

Cases are patients with more than one episode of venous thromboembolism, whereas controls have experienced only one VTE episode

^a^Crude value

^b^Adjusted for age, gender, and malignancy at time of sampling

### FVIII:C

Median FVIII:C was higher in cases than in controls, with values of 1.8 IU/ml (2.5–97.5 percentile 0.9–3.4) versus 1.6 IU/ml (2.5–97.5 percentile 0.7–2.8), respectively (*p* < 0.001). Controls in whom the index event was unprovoked had a higher FVIII:C than controls in whom the index event was provoked by a risk factor for VTE, with median values of 1.8 IU/ml (2.5–97.5 percentiles 0.9–2.9) and 1.6 IU/ml (2.5–97.5 percentiles 0.7–3.0), respectively (*p* = 0.001). Compared with the reference group (i.e. those in the lowest quintile of FVIII:C), patients in the highest quintile were at increased risk of recurrent VTE with an OR of 5.4 (95 % CI 1.9–15.1) (Table 3). The risk of recurrence was slightly lower after adjustment for age, gender and active malignancy at time of sampling, with an OR of 4.2 (95 % CI 1.4–12.2). Patients with the predefined threshold of FVIII:C > 1.66 IU/ml were at increased risk of recurrent VTE, with a crude OR of 1.6 (95 % CI 1.0–2.7). This increased risk was slightly adjusted towards unity after correction for age, gender, active malignancy at time of sampling (OR 1.4, 95 % CI 0.8–2.4).

**Table 3:** Factor VIII activity in cases and controls

Time to index PE	Percentile	FVIII:C (IU/ml)	Cases (*N* = 72)	Controls (*N* = 277)	OR^a^ (95 % CI)	OR^b^ (95 % CI)
All patients	0–20	0–1.20	5	57	Ref	Ref
	20–40	1.20–1.48	14	54	3.0 (1.0–8.8)	2.7 (0.9–8.1)
	40–60	1.48–1.74	15	54	3.2 (1.1–9.3)	2.7 (0.9–8.1)
	60–80	1.74–2.02	13	57	2.6 (0.9–7.8)	2.3 (0.8–7.1)
	80–100	>2.02	25	53	5.4 (1.9–15.1)	4.2 (1.4–12.2)
0–2 years	0–20	0–1.20	1	9	Ref	Ref
	20–40	1.20–1.48	2	10	1.8 (0.1–23.4)	1.5 (0.1–22.4)
	40–60	1.48–1.74	2	13	1.4 (0.1–17.7)	1.2 (0.1–16.8)
	60–80	1.74–2.02	2	12	1.5 (0.1–19.2)	0.7 (0.0–12.5)
	80–100	>2.02	3	13	2.1 (0.2–23.3)	1.4 (0.1–18.2)
2–4 years	0–20	0–1.20	4	22	Ref	Ref
	20–40	1.20–1.48	4	19	1.2 (0.2–5.3)	1.3 (0.3–6.1)
	40–60	1.48–1.74	6	17	1.9 (0.5–8.0)	2.0 (0.4–8.8)
	60–80	1.74–2.02	6	22	1.5 (0.4–6.1)	1.7 (0.4–7.5)
	80–100	>2.02	11	22	2.8 (0.8–10.0)	2.7 (0.7–11.0)
>4 years	0–20	0–1.20	0	26	Ref	Ref
	20–40	1.20–1.48	8	25	Infinite	Infinite
	40–60	1.48–1.74	7	24	Infinite	Infinite
	60–80	1.74–2.02	5	23	Infinite	Infinite
	80–100	>2.02	11	18	Infinite	Infinite

Cases are patients with more than one episode of venous thromboembolism, whereas controls have experienced only one VTE episode

^a^Crude value

^b^Adjusted for age, gender, and malignancy at time of sampling

In the regression analysis stratified for time between index PE and study visit, no effect of time on the relative risks of recurrent VTE comparing the different quintiles with the reference group was observed. However, none of the patients in the reference group in whom time between index PE and study visit exceeded three years developed a recurrent VTE. Therefore, relative risks of recurrent VTE for the longest time stratum (>4 years between index PE and the study visit) could not be computed.

## Discussion

This study, in which we assessed the association between MP-TF activity, FVIII:C and recurrent VTE, has two main findings. First, there was no association between MP-TF activity and the risk of VTE recurrences. Second, high FVIII:C levels were associated with a 4-fold increased risk of VTE recurrence. This finding was consistent throughout the different strata of time between index PE and study visit.

Until now, the role of MP-TF in VTE pathogenesis has predominantly been studied in cancer patients, for whom the risk of thromboembolic disease is markedly increased [24, 25]. In patients with malignancy, MP-TF has been proposed as a key link between cancer progression and cancer-associated thrombosis, which are known to be strongly connected [26]. However, studies assessing the potential of MP-TF to predict VTE in cancer patients yielded conflicting results. Although the majority of studies on this topic reported high MP-TF levels or high MP-TF activity to be associated with an increased risk of VTE [23, 27–29], two other studies could not confirm this association [30, 31].

Studies evaluating the role of MP-TF in the pathophysiology of VTE in the general (non-cancer) population are scarce and so far no study specifically addressed its association with recurrent VTE. Two previous studies reported that MP-TF activity was not increased in patients with a first manifestation of acute VTE compared to control patients [13, 32]. In contrast, Ye et al. did observe higher monocyte-derived MP-TF levels and MP-TF activity in patients with an acute episode of first or recurrent DVT compared with healthy controls although this study comprised only 50 patients [14]. Moreover, compared with healthy controls, the number of platelet and endothelial derived MP-TF was only increased in those with recurrent DVT, not in patients with a first DVT. The discrepancy throughout the literature on the association between MP-TF levels or activity and VTE might be a result of differences in methods and timing of blood sampling (i.e. duration from the VTE event), patient setting (cancer-patients or general VTE population), the absence of a standardized assay to measure MP-TF, and the fact that detection of MPs is cumbersome due to their small size [33]. Based on the existing literature and the results of our study, being the largest in this setting up to date, the association between MP-TF activity and recurrent VTE in the general VTE population appears to be weak or non-existent.

It has well been established that factors associated with a first episode of VTE may not necessarily be involved with the development of recurrent events [34]. Whether this scenario holds for FVIII:C is currently unclear. Several studies conclusively found associations between FVIII:C and a first manifestation of VTE [18, 35, 36], whereas studies on FVIII:C and recurrent VTE have shown conflicting results [15–20]. Four out of the six studies in this setting demonstrated a positive association between FVIII:C and the risk of recurrent VTE [16–19]. However, the large Leiden Thrombophilia study (LETS) could not confirm this association [15]. Notably, this study used a predefined cut-off of 1.66 IU/ml for the definition of elevated FVIII:C, rather than studying percentiles of FVIII:C [35]. The Prevention of Recurrent VTE(PREVENT) trial was also unable to find an association between elevated FVIII:C and recurrent VTE, although a non-significant trend towards a higher recurrence risk for patients with elevated FVIII:C was noted (21 vs. 13 %, *p* = 0.18) [20]. In an attempt to provide more clarity on this topic, we used the data of a large sample of patients with acute PE. We did not observe an association between recurrent VTE and increased FVIII:C at the cut-off of 1.66 IU/ml, consistent with the LETS study. However, when FVIII:C was divided in quintiles, a 4-fold increased risk of VTE recurrence was found for patients with FVIII:C above the 80th percentile, thus supporting the studies that found a positive association between FVIII:C and the risk of recurrent VTE.

This study has several strengths and limitations. The fact that, in line with numerous previous reports, male gender and unprovoked PE were identified as important determinants for recurrent VTE, supports the representativeness of our cohort. In addition most studies performed so far only included patients with DVT, whereas the present study focusses on patients with PE, and therefore, our study provides new information to the literature. Furthermore, we studied MP-TF and FVIII activity at least one year after PE diagnosis, ruling out the initial effect of acute PE on the test results. An important limitation of this study includes the fact that a significant proportion of patients had died before they could be included in the study, thereby enhancing the risk of survival bias. Moreover, of the 588 patients alive at time of enrolment, 148 were unavailable for objective testing on pulmonary hypertension and therefore not included. We cannot exclude that is has affected our study outcomes, in a worst-case scenario, underestimating the association between MP-TF and recurrent VTE. Second, as a consequence of its design, this study is unable to assess whether MP-TF or FVIII activity are predictive for recurrent VTE. Third, blood samples were drawn at a various duration of follow-up after the index event. Nonetheless, our regression analyses stratified for time between index PE and study enrollment did not demonstrate an effect of duration of this time period on the association between MP-TF or FVIII:C and recurrent VTE. Fourth, MP-TF activity was assessed once in every patient, whereas MP-TF activity levels are known to fluctuate. However, it is unlikely that this fluctuation would be different for cases and controls, thereby influencing our study results on the association between MP-TF activity and recurrent VTE. Yet, we recognize that further prospective studies are needed to evaluate the association between MP-TF activity in both the acute and chronic phase of established thromboembolic disease and recurrent VTE.

To summarize, in our study population MP-TF does not appear to play an important role in driving the risk for VTE recurrences. Also considering the difficulty of this assay, and its unavailability in most hospitals, implementation of MP-TF in prognostic models for risk stratification does not seem warranted. Additionally, our data provide supporting evidence to consider elevated FVIII:C as a marker for a hypercoagulable state and as a contributing factor for the recurrence of VTE. Future prospective studies are necessary to explore its potential as a tool for prognostication, either by itself or in combination with other pro-thrombotic markers.
